# A characterization of cross-border use of health services in a transborder population at the Mexico-Guatemala border, September–November 2021

**DOI:** 10.1371/journal.pone.0282095

**Published:** 2023-02-22

**Authors:** Cesar Rodriguez-Chavez, Silvana Larrea-Schiavon, Rene Leyva-Flores, Nirma D. Bustamante, Marcel Arevalo, Ricardo Cortes-Alcala, Georgina Rodriguez, Rebecca Merrill, Dianne Escotto, Ietza Bojorquez

**Affiliations:** 1 School of Public Health, University of Texas, Health Science Center, Houston, Texas, United States of America; 2 School of Public Health, University of California Berkeley, Berkeley, California, United States of America; 3 Center for Research on Health Systems, Instituto Nacional de Salud Pública, Cuernavaca, Morelos, México; 4 U.S. Centers for Disease Control and Prevention, Mexico City, Mexico; 5 Program on Migration and Poverty, Facultad Latinoamericana de Ciencias Sociales (FLACSO)–Guatemala, Guatemala, Guatemala; 6 General Directorate for Health Promotion, Ministry of Health, Mexico City, Mexico; 7 U.S. Centers for Disease Control and Prevention, Atlanta, GA, United States of America; 8 Department of Population Studies, El Colegio de la Frontera Norte, Tijuana, Baja California, Mexico; National Institute of Public Health: Instituto Nacional de Salud Publica, MEXICO

## Abstract

**Background:**

Cross-border use of health services is an important aspect of life in border regions. Little is known about the cross-border use of health services in neighboring low- and middle-income countries. Understanding use of health services in contexts of high cross-border mobility, such as at the Mexico-Guatemala border, is crucial for national health systems planning. This article aims to describe the characteristics of the cross-border use of health care services by transborder populations at the Mexico-Guatemala border, as well as the sociodemographic and health-related variables associated with use.

**Methods:**

Between September-November 2021, we conducted a cross-sectional survey using a probability (time-venue) sampling design at the Mexico-Guatemala border. We conducted a descriptive analysis of cross-border use of health services and assessed the association of use with sociodemographic and mobility characteristics by means of logistic regressions.

**Results:**

A total of 6,991 participants were included in this analysis; 82.9% were Guatemalans living in Guatemala, 9.2% were Guatemalans living in Mexico, 7.8% were Mexicans living in Mexico, and 0.16% were Mexicans living in Guatemala. 2.6% of all participants reported having a health problem in the past two weeks, of whom 58.1% received care. Guatemalans living in Guatemala were the only group reporting cross-border use of health services. In multivariate analyses, Guatemalans living in Guatemala working in Mexico (compared to not working in Mexico) (OR 3.45; 95% CI 1.02,11.65), and working in agriculture/cattle, industry, or construction while in Mexico (compared to working in other sectors) (OR 26.67; 95% CI 1.97,360.85), were associated with cross-border use.

**Conclusions:**

Cross-border use of health services in this region is related to transborder work (i.e., circumstantial use of cross-border health services). This points to the importance of considering the health needs of migrant workers in Mexican health policies and developing strategies to facilitate and increase their access to health services.

## Introduction

Cross-border use of health services consists of the movement of persons in order to receive care in a different country [1] and is mostly driven by a) distance, when the services in the other country are closer to the place of residence; b) familiarity, when the user feels more culturally identified or has better knowledge of services in the other country; c) cost, when prices in the other country are lower for the same or similar services; and d) specialized care, when a service needed is not available in one’s own side of the border [1, 2].

The cross-border use of private health services can improve the economy of the country where those services are provided [1]. When cross-border use is directed toward public health services, the situation might seem different, since people would be using publicly financed services intended and planned for the local population. However, from a public health perspective, providing essential services in border areas can protect the health of populations that have constant interaction.

Cross-border use of health services has been studied mostly as movement from high- to low- and middle-income countries, but other types of transnational health care seeking are possible, including the use of services in neighboring countries of similar income, or from less to more developed countries [1, 3, 4]. In the American continent, the majority of the public health literature on this matter refers to the use of services in the United States (U.S.)-Mexico border, where people living in the former country seek care in the latter. In this case, reasons for seeking care abroad include differences in costs, limited coverage of some services under health insurance systems in the U.S., and, for people in the U.S. who identify as Mexican, cultural identity and familiarity with services [5–9]. Given the higher health costs and regulations for entrance to the U.S., cross-border use of healthcare services by Mexican residents is less common.

The literature on cross-border use in other parts of the Americas is scarce, but there are reports of use driven by geographical proximity in the Haiti-Dominican Republic border [2], and by a combination of perceived quality, geographical availability, and the advantages that can be accrued by having a child born in a different country in the case of maternal care in the Amazonian border between Brazil, Peru and Colombia [10]. The cross-border use of health services at the Mexico-Guatemala border is poorly characterized, but previous articles have reported on maternal health services use in the region [11], and the use of services in Mexico by residents of a Guatemalan area where health services are more difficult to access than in the Mexican side [12].

The border region between Mexico and Guatemala shares cultural, economic, social, and historical links that date back to pre-Columbian times [13]. Over two million people live along this border, with seven land points of entry/exit officially recognized by the authorities of both countries and over 100 irregular crossing points [14–17]. In 2021, over 60,000 Guatemalans were registered in Mexico either as regional visitors or as border workers [18], and an unspecified number of irregular border crossers add to the binational movement. Of the Guatemalans returning through the land border after visiting Mexico, the majority had been in Mexico for only a limited period (39.1% up to 24 hours, and 54.9% from one to 30 days in 2019) [19]. At the national level, Guatemalan migrants represent 5.6% of all international migrants in Mexico, but they make up 93% of immigrants in municipalities on the southern border of Mexico [14]. Additionally, 97% of the Guatemalan migrants entering Mexico come from the border region of Guatemala [14], contributing to the intensity and frequency of transborder crossing of people at the Guatemala-Mexico border.

Due to the implications for public health of these population dynamics, like the risk of communicable disease transmission, this team implemented a survey from September to November 2021 evaluating health practices of transborder crossers and aspects related to the COVID-19 pandemic at the Mexico–Guatemala border. Given the public health implications and the large gap in the literature describing this critical international border, the aim of this article is to describe the characteristics of the cross-border use of healthcare services by transborder populations at the Mexico-Guatemala border, as well as the sociodemographic and health-related variables associated with such use.

## Methods

This was a cross-sectional survey conducted from September 15 to November 15, 2021 in three cities on the Guatemalan side of the border (El Carmen, La Mesilla, and Tecún Umán), representing the main points of entry on the shared border (98% of all registered land arrivals in the Mexican state of Chiapas occur via these three Guatemala cities) [16, 18]. The survey’s methods have been described in detail in a previous article describing COVID-19 vaccination and acceptance [20].

### Design and sample

The survey was conducted using a time-venue sampling design. Following the methodology used in Mexico´s Survey of Migration in the Southern Border (EMIF-Sur) (www.colef.mx/emif), the design is based on the events of border crossings in a given time period. As such, one person could be counted more than once if they crossed the border several times during the study period. For this study, combinations of 8-hour periods within days of the week (time component) and cities and specific locations of data collection (venue component) were used to construct the sampling frame. Combinations of the time and venue components (strata) were then selected based on a probability proportional to the number of crossings. The number of crossings was then used to calculate sampling weights.

The data collection locations were where people congregate after crossing the border (mainly by foot), such as bus and taxi terminals that are mostly located a few meters after the crossing point. In those locations, the interviewers defined an imaginary line, and approached consecutive persons that crossed that line to obtain consent, assess eligibility, and apply the survey in tablets. The mean time to completion of the questionnaire was 3.4 minutes. Participants did not receive any compensation for responding the questionnaire.

### Participants

The study participants were persons who had crossed the border into Guatemala from Mexico. The inclusion criteria were: 1) 18 years of age or older, 2) having crossed into Guatemala in the past 12 hours, and 3) giving informed consent to participate. We limited the sample to persons born and living in Guatemala, persons born in Guatemala and living in Mexico, persons born and living in Mexico, and persons born in Mexico and living in Guatemala. Participants were excluded from the survey if they did not provide enough information to assess eligibility, did not give their consent to participate, or were not born or living in Guatemala or Mexico.

### Questionnaire design

The questionnaire applied in the survey was comprised of 34 questions that addressed five main dimensions: 1) Filter questions to assess eligibility, 2) Sociodemographic characteristics, 3) Frequency and motives of border crossing, 4) Health status and use of health services, and 5) COVID-19 knowledge, behaviors, history of disease, vaccination, and attitudes towards vaccination.

### Outcomes

The main outcome was cross-border use of healthcare services. In order to assess it, we followed two different strategies. First, we considered as cross-border users of health care services those participants who stated that their main reason for entering Guatemala, or for having crossed to Mexico, was “to visit the doctor, buy medicines, or some other health-related reason”. Second, we asked participants if, in the past two weeks, they had experienced any health problems. If they answered yes, we asked if they had received care in a health service for their most recent health problem. If they did, we asked in what country they had received health care (Mexico, Guatemala, or another country), and defined cross-border use as the use of health services in one country (Guatemala or Mexico) by a person living in the other country for more than one year, regardless of country of birth. For this second strategy, we also asked about the type of healthcare facility visited (public health center/hospital, private health center/hospital, pharmacy, international organization or agency, civil society organization, or another place).

### Independent variables

To evaluate the association of sociodemographic and health-related characteristics with cross-border healthcare use, we classified participants into four mobility groups based on country of birth and residence: Guatemalans living in Guatemala (G-G), Guatemalans living in Mexico for at least a year (G-M), Mexicans living in Mexico (M-M), and Mexicans living in Guatemala for at least a year (M-G). The groups were defined using three variables: self-report of the country of birth, self-report of the country of residence, and time spent on their last visit to Guatemala or Mexico. For instance, a person born and living in Guatemala who had spent less than one year in Mexico on their last visit to this country was categorized as a G-G. However, a person who self-reported having been born and living in Guatemala but who spent more than one year in Mexico during their last visit to this country was categorized as a G-M.

Other independent variables included in the analysis were age group, sex, ethnicity (indigenous or not indigenous), educational level (none, elementary school, middle school, high school or technical school, and Bachelor´s degree or higher), time spent in Mexico in the last visit (as a continuous variable measured in days), job status in their last visit to Mexico (worked or not worked), type of work performed in Mexico (agriculture/cattle, industry/manufacturer, construction, merchant, informal commerce, domestic services, diverse services, and professionals), and most recent health problem (wounds/accidents, not COVID-related respiratory diseases, gastrointestinal diseases, pregnancy or delivery care, family planning, vector-borne diseases, COVID-19, and other diseases). Because of the small sample size in some combinations of variables, we decided to dichotomize those with multiple answer choices. Educational level was categorized as at least some formal education (defined by those who reported an education level of elementary school or higher) vs. no formal education. The type of work performed in Mexico was categorized as agricultural/livestock, industrial, or construction workers vs. workers in all other sectors unrelated to the latter. Most recent health problems were categorized as wounds/accidents vs. other health problems.

### Analysis

We conducted a descriptive analysis of sociodemographic characteristics, mobility characteristics, the prevalence of health problems, use of health services, and cross-border use of health services, across the whole sample and by groups of the country of birth and residence. We report the data as means or percentages and 95% confidence intervals. We then explored the bivariate association between cross-border use of health services and the independent variables. We performed bivariate and multivariable analyses only among G-G because the unweighted number of G-M, M-M, and M-G that had used health services due to a recent health problem was too small to analyze as separate groups.

For the multivariable analysis, we used logistic regression to evaluate the association between cross-border use of health services and the independent variables. Following a theory-based approach, all independent variables were kept in the model, regardless of the significance of the association. In addition, given that type of work was associated with the cross-border use of health services in the bivariate analysis, we employed a second regression model to evaluate the multivariate association between the type of work and cross-border use of services.

All analyses considered the sampling design (weights, strata, and clusters) and were done in Stata 17.0 (StataCorp), using the SVY module.

### Ethical considerations

We followed an informed consent process, explaining the aims and procedures of the study to participants. The interviewers read an informed consent script, describing the study’s aims, procedures, potential risks and benefits, data confidentiality and the voluntary nature of participation, and obtained verbal consent from all participants. No personal, identifiable data were collected from participants as part of this survey. The ethics committee of El Colegio de la Frontera Norte reviewed and approved the survey protocol [079_230821], including the verbal consent. This activity was deemed not to be research as defined in 45 CFR 46.102(l) and an IRB review was not required by the U.S. Centers for Disease Control and Prevention.

## Results

**Fig 1:**
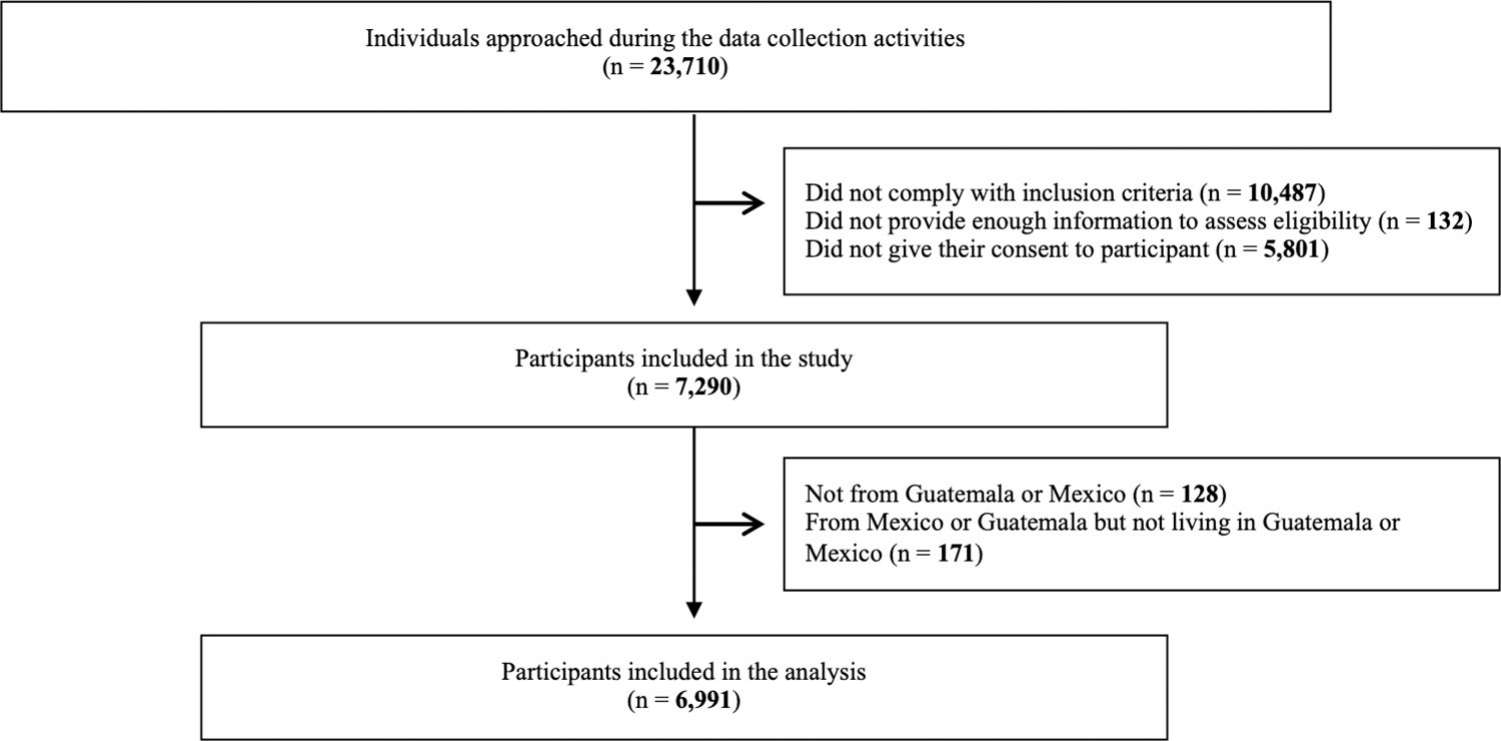
Flowchart of the selection of study participants.

The total number of persons approached was 23,710. Of them, 10,487 (44.2%) did not fulfill the inclusion criteria for the study, 132 (0.6%) did not provide enough information to assess eligibility, and 5,801 (24.5%) did not give their consent to participate, resulting in 7,290 participants who consented to participate. For this analysis, we included only those that were from Guatemala or Mexico and were living in either of these countries, giving a total of 6,991 (95.9% of the consented participants) participants, which, after applying sampling weights, represented 160,540 crossing events (Fig 1).

### Sociodemographic and cross-border mobility characteristics

Table 1 describes the sociodemographic characteristics of survey participants. Of the total participants included in this analysis, 82.9% (95% CI 81.0,84.6) were G-G, 9.2% (95% CI 8.3,10.2) G-M, 7.8% (95% CI 6.7,9.0) M-M, and 0.16% (95% CI 0.05,0.5) M-G. The mean age across all participants was 36 years (95% CI 35.6, 36.5). M-M were older than G-G and G-M. Mobility groups also differed regarding sex, ethnicity, and educational level, while religious affiliation showed similar proportions. People self-identifying as indigenous were mostly from G-G (43.3%; 95% CI 40.4,46.2). Guatemalans, independent of country of residence, had the lowest education level.

**Table 1:** Sociodemographic characteristics of survey participants.

	Mobility group				
	Guatemalans living in Guatemala (G-G)	Guatemalans living in Mexico (G-M)	Mexicans living in Mexico (M-M)	Mexicans living in Guatemala (M-G)	Total
	n = 5,773	n = 654	n = 557	n = 7	n = 6,991
**Participants** % (95%CI)^1^	82.9% (81.0, 84.6)	9.2% (8.3, 10.2)	7.8% (6.7, 9.0)	0.16% (0.06, 0.5)	**100%**
**Age group** % (95% CI)					
18–29 y	33.4 (31.6,35.2)	30.8 (26.2,35.9)	23.1 (19.1,27.8)	60.3 (38.2,78.9)	**32.4** **(30.7,34.1)**
30–64 y	65.5 (63.8,67.3)	66.4 (61.2,71.2)	68.4 (61.9,74.3)	39.7 (21.1,61.8)	**65.8** **(64.2,67.5)**
65+ y	1.0 (0.8,1.4)	2.8 (1.8.4.4)	8.4 (5.6,12.6)	0	**1.8** **(1.4,2.2)**
**Female** % (95% CI)	31.9 (29.7,34.1)	50.4 (45.5,55.3)	48.3 (42.6,54.1)	29.7 (4.9,77.4)	**34.8** **(32.8,36.9)**
**Religion** % (95% CI)					
Catholic	39.7 (37.3,42.2)	34.6 (29.7,39.9)	37.5 (32.0,43.4)	37.1 (12.4,71.0)	**39.1** **(36.9,41.4)**
Non-Catholic Christian or another	42.7 (39.4,46.1)	42.7 (36.8,48.9)	35.7 (30.2,41.7)	44.6 (22.1,69.5)	**42.2** **(39.2,45.2)**
None	17.6 (15.1,20.4)	22.7 (18.1,28.1)	26.7 (21.5,32.8)	18.3 (2.0,70.7)	**18.7** **(16.3,21.3)**
**Ethnicity** % (95% CI)					
Indigenous	43.3 (40.4,46.2)	19.2 (14.6,24.9)	0.9 (0.1,5.1)	2.0 (0.3,12.6)	**38.0** **(35.3,40.7)**
Afro-descendant	0.7 (0.4,1.2)	0.3 (0.08,1.1)	0.3 (0.04,2.1)	0	**0.6** **(0.4,1.1)**
**Educational level** % (95% CI)					
None	13.3 (11.4,15.4)	17.8 (14.3,21.9)	14.4 (11.3,18.2)	0	**13.8** **(12.1,15.7)**
Elementary school	59.2 (56.8,61.6)	51.1 (46.5,55.8)	38.7 (33.1,44.7)	20.2 (3.6,63.4)	**56.9** **(54.8,59.0)**
Middle school	21.3 (19.4,23.4)	19.4 (15.9,23.4)	28.8 (24.5,33.6)	61.5 (15.3,93.4)	**21.8** **(20.1,23.6)**
High school | Technical school	5.5 (4.8,6.2)	9.1 (6.5,12.4)	10.0 (7.5,13.1)	0	**6.1** **(5.5,6.9)**
Bachelor’s degree or higher	0.7 (0.5,1.0)	2.6 (1.3,5.0)	8.0 (5.7,11.2)	18.3 (2.0,70.7)	**1.4** **(1.2,1.8)**
**Worked in their last stay in Mexico** % (95% CI)	58.9 (56.3,61.4)	47.5 (41.5,53.5)	32.2 (27.8,36.8)	27.2 (3.7,78.3)	**56.1** **(53.7,58.5)**
**Work performed in Mexico** % (95% CI)					
Agriculture/Livestock	43.6 (40.5,46.7)	26.2 (19.0,35.0)	10.2 (6.0,16.8)	27.3 (2.2,86.2)	**41.4** **(38.5,44.4)**
Industry/Manufacturer	5.7 (4.7,6.9)	2.9 (1.6,5.3)	9.7 (6.0,15.4)	0	**5.7** **(4.8,6.9)**
Construction	13.9 (11.6,16.5)	6.6 (4.0,10.6)	7.9 (4.1,14.6)	0	**13.3** **(11.2,15.7)**
Merchant	13.9 (11.7,16.5)	14.6 (9.7,21.4)	33.6 (24.2,44.6)	72.7 (13.8,97.8)	**14.7** **(12.6,17.1)**
Informal commerce	2.9 (2.3,3.6)	5.7 (3.2,9.9)	3.5 (1.3,8.7)	0	**3.0** **(2.4,3.8)**
Domestic services	6.6 (5.7,7.7)	19.3 (11.7,30.1)	6.0 (2.4,14.5)	0	**7.2** **(6.2,8.3)**
Diverse services	9.1 (8.0,10.3)	12.7 (8.1,19.3)	14.2 (8.9,21.9)	0	**9.4** **(8.4,10.6)**
Professionals	4.4 (3.5,5.4)	12.1 (7.0,20.0)	14.9 (9.3,23.0)	0	**5.1** **(4.2,6.2)**
**The type of work performed was either in agriculture/livestock, industry, or construction** % (95% CI)	63.2 (60.0,66.3)	35.7 (28.3,43.9)	27.9 (20.8,36.2)	27.3 (2.2,86.2)	**60.5** **(57.5,63.3)**

^1^ Percentages and CIs consider sampling design

As for work-related variables, more than half of all participants worked in Mexico the last time they were in that country (56.1; 95% CI 53.7, 58.5). A significantly higher percentage of G-G reported working in Mexico (58.9%; 95% CI 56.3,61.4), compared to G-M (47.5%; 95% CI 41.5,53.5), and M-M (32.2%; 95% CI 27.8,36.8). The type of work in Mexico differed by mobility group and sex: 43.6% of G-G (95% CI 40.5,46.7%) and 26.2% of G-M (95% CI 6.0,16.8) were agriculture/livestock workers, compared to 10.2% of M-M (95% CI 6.0,16.8). Most G-G and G-M women worked in domestic services (30.2% [95% CI 26.3,34.4] and 40.4% [95% CI 26.2,56.5], respectively), while most M-M women worked as merchants (50.1%; 95% CI 33.7,66.4) (results not shown). Since there were only seven participants in the M-G group, and none reported a recent health problem, this group was excluded from analyses, except for the description of their sociodemographic characteristics in Table 1.

In Table 2, we present the frequency of crossing and days spent in Mexico during their last visit for participants in the G-G group. The question about the frequency of crossing wasn’t asked of participants living in Mexico, either G-M or M-M. Most reported crossing to Mexico from Guatemala at least once every month (56.2%; 95% CI 53.2,59.2). The mean number of days of stay in Mexico among all participants was 47.3 (95% CI 44.3,50.3). As for time spent in Mexico by job status, 62.6% (95% CI 58.4,66.6) of G-G who worked during their last trip to Mexico reported staying for more than one month, compared to 6.3% (95% CI 4.6,8.6) of those who did not work, and among G-G who worked during their last trip to Mexico, the mean number of days they stayed in Mexico was 71.8 days (95% CI 67.7,75.8), compared to 12.4 days (95% CI 10.6,14.1) among those who did not work (Table 2).

**Table 2:** Mobility characteristics of Guatemalans living in Guatemala, by job status.

	Job status		Total
	Worked during their last trip to Mexico	Did not work on their last trip to Mexico	
	n = 3,246	n = 2,527	n = 5,773
**Frequency of crossings to Mexico** % (95% CI)^1^			
Daily	1.8 (1.3,2.6)	7.5 (5.4,10.3)	**4.0** **(3.1,5.3)**
At least once a month	62.9 (59.4,66.3)	45.7 (40.9,50.7)	**56.2** **(53.2,59.2)**
Less than once a month	35.2 (32.0,38.6)	46.7 (42.6,50.9)	**39.7** **(37.1,42.4)**
**Time spent in Mexico during their last visit** % (95% CI)			
One day or less	11.0 (9.0,13.4)	39.9 (35.8,44.0)	**22.9** **(20.4,25.6)**
Between two days and two weeks	6.9 (5.5,8.6)	37.0 (32.9,41.2)	**19.3** **(17.2,21.5)**
Between >two weeks and one month	19.5 (16.9,22.3)	16.8 (14.2,19.8)	**18.4** **(16.4,20.5)**
More than one month	62.6 (58.4,66.6)	6.3 (4.6,8.6)	**39.5** **(36.6,42.4)**
**Mean number of days spent in Mexico on their last visit** mean (95% CI)	71.8 (67.7,75.8)	12.4 (10.6,14.1)	**47.3** **(44.3,50.3)**

^1^ Percentages and Cis consider sampling design

### Health problems and use of health services

Table 3 describes the health problems in the two weeks prior to taking the survey and the use of health services, by mobility group. As in Table 2, M-G were not included in this analysis since there were only seven participants in this group and none of them reported a recent health problem.

**Table 3:** Health problems in the last two weeks and use of health services, by mobility group.

	Mobility group			
	Guatemalans living in Guatemala (G-G)	Guatemalans living in Mexico (G-M)	Mexicans living in Mexico (M-M)	Total
	n = 5,773	n = 654	n = 557	n = 6,991
**Presence of a health problem** % (95% CI)^1^	2.7 (2.1,3.6)	4.1 (2.3,7.3)	0.6 (0.2,1.7)	**2.6** **(2.0,3.4)**
	**n = 170**	**n = 14**	**n = 4**	**n = 188**
**Most recent health problem** % (95% CI)^2^				
Wounds and accidents	53.6 (43.2,63.7)	31.6 (12.4,60.0)	59.0 (11.2,94.2)	**52.2** **(42.6,61.6)**
Respiratory diseases (not COVID-related)	22.7 (13.6,35.5)	30.4 (7.6,70.0)	0	**22.9** **(14.2,34.6)**
Gastrointestinal diseases	19.8 (13.2,28.5)	6.7 (0.6,44.9)	26.1 (2.0,85.6)	**19.0** **(12.9,27.1)**
Pregnancy or delivery care	0.9 (0.2,4.5)	0	0	**0.8** **(0.2,4.1)**
Family planning	0.8 (0.08,7.2)	0	0	**0.7** **(0.07,6.7)**
Vector-borne diseases	1.0 (0.2,6.7)	12.1 (1.9,49.4)	0	**1.8** **(0.5,6.1)**
COVID-19	0.8 (0.09,7.5)	10.8 (1.2,54.5)	0	**1.5** **(0.3,6.7)**
Other	0.4 (0.04,3.7)	8.4 (0.8,51.2)	14.9 (1.0,74.4)	**1.2** **(0.3,4.6)**
**Received care for the most recent health need** % (95% CI)^2^	57.1 (46.7,67.0)	68.3 (35.3,89.5)	69.6 (14.4,96.9)	**58.1** **(48.4,67.2)**
	n = 97	n = 10	n = 3	**n = 117**
**They received care in Mexico**% (95% CI)^2^	65.4 (52.9,76.1)	100	100	**69.0** **(57.6,78.5)**
**Type of facility where they received** **Healthcare services** % (95% CI)^3^				
Public primary care clinic/hospital	59.1 (43.7,72.8)	35.3 (7.3,79.1)	58.9 (5.6,97.2)	**57.3** **(43.1,70.4)**
Private clinic/hospital	11.4 (4.9,24.5)	8.2 (0.7,54.3)	41.1 (2.8,94.4)	**11.8** **(5.1,24.9)**
Pharmacy	17.8 (8.8,32.5)	48.1 (15.8,82.0)	0	**19.7** **(11.1,32.5)**
International organization or agency	7.8 (3.2,17.7)	8.4 (0.7,54.8)	0	**7.7** **(3.4,16.3)**
Civil society organization	4.0 (1.1,13.5)	0	0	**3.6** **(0.8,12.2)**

^1^ Percentages and Cis consider sampling design

^2^ Percentage calculated over those who reported a health problem in the past two weeks.

^3^ Percentages were calculated over those who received care for their most recent care need.

**Fig 2:**
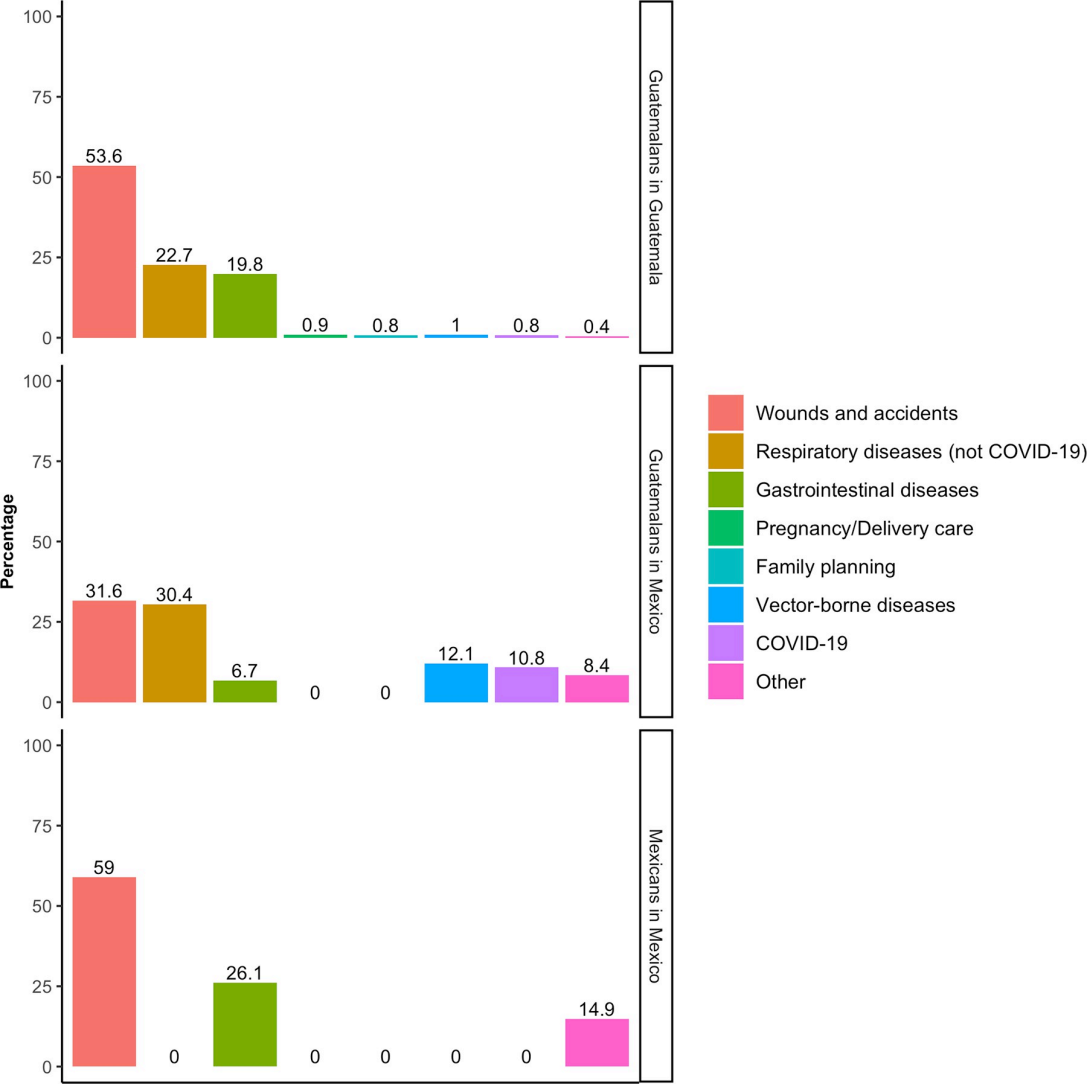
Most recent health problem, by mobility group.

Of the total participants, 2.6% (95% CI 2.0,3.4) had a recent health problem, with a lower prevalence among M-M (0.6%; 95% CI 0.2,1.7), compared to G-M (4.1%; 95% CI 2.3,7.3), and G-G (2.7%; 95% CI 2.1,3.6). The most frequent health problem reported across all groups were wounds and accidents (52.2%; 95% CI 42.6,61.6) (Fig 2), and 44.3% of participants who had a health problem in the past two weeks reported communicable diseases (respiratory, gastrointestinal, vector-borne, and COVID-19) as their most recent health problem.

**Fig 3:**
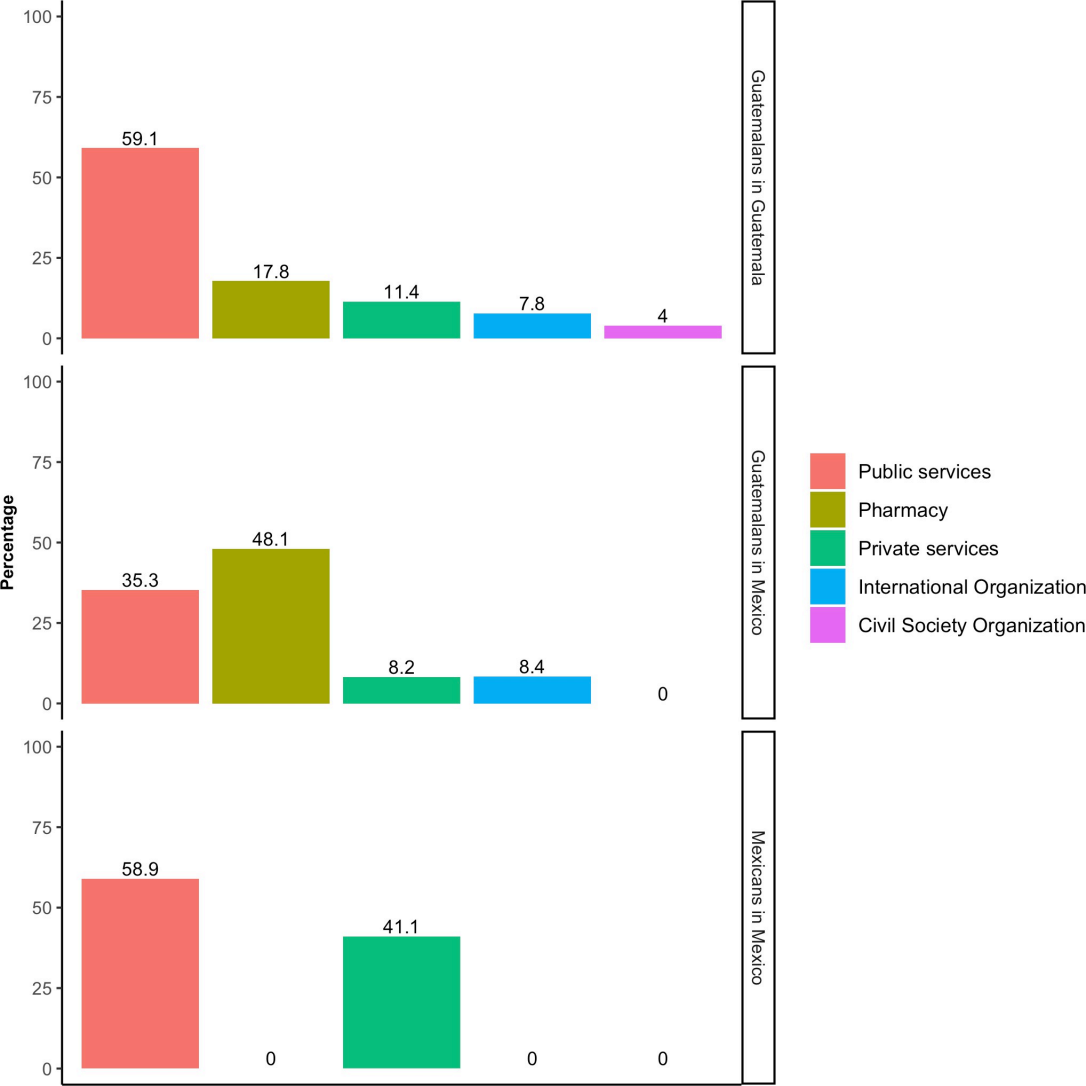
Type of health service used, by mobility group.

Of those participants that had experienced a health problem in the previous two weeks (unweighted n = 188), 57.1% of G-G (95% CI 46.7,67.0), 68.3% of G-M (95% CI 35.3,89.5), and 69.6% of M-M (95% CI 14.4,96.9) received care, and the majority had received care in Mexico. Public primary care clinics and hospitals were the main types of facilities where participants received care (57.3%; 95% CI 43.1,70.4). M-M reported receiving care mostly in public or private clinics and hospitals, while the services used by Guatemalans included pharmacies and civil society services (Table 3, Fig 3).

### Cross-border use of health services

**Fig 4:**
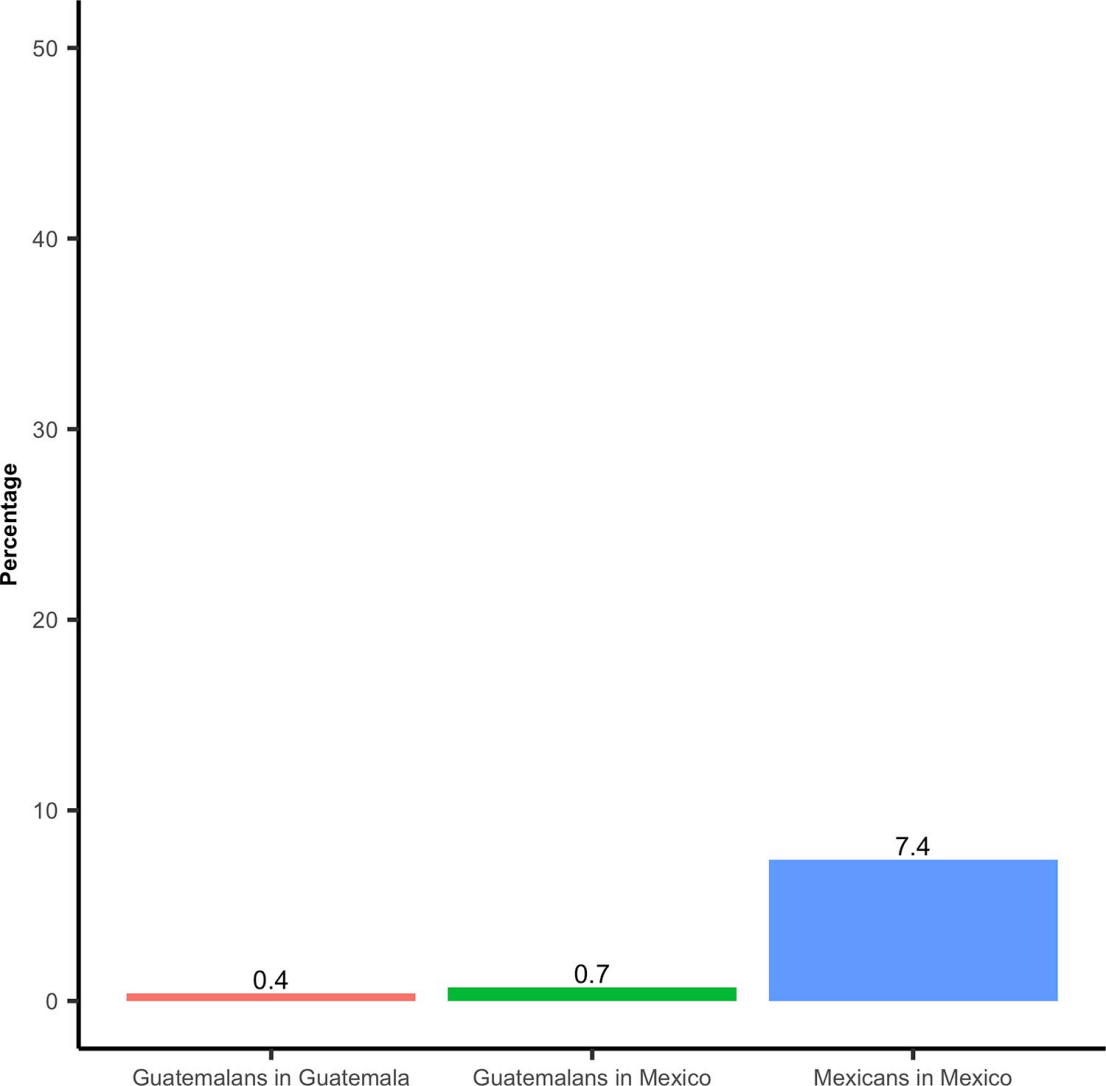
The reason for the last cross to a country where they were not living in was health-related, by mobility group.

A minority of participants (1.0%; 95% CI 0.7,1.4) stated the main reason for their last cross to the neighboring country was health-related (i.e., going to the doctor, buying medicines, or other health-related reasons). This percentage was higher among women (1.8%; 95% CI 1.1,2.8) than men (0.6%;95% CI 0.4,0.9) (results not shown), and among M-M (7.4%; 95% CI 5.0,10.8), compared to G-M (0.7%; 95% CI 0.3,2.2), and G-G (0.4%; 95% CI 0.3,0.7) (Fig 4).

Among participants that received care for their most recent health problem (n = 117), none of the G-M or M-M received care in Guatemala, while 65.4% (95% CI 52.9,76.1) of G-G received care in Mexico. Most of the G-G who received care in Mexico went to public healthcare services (45.0%; 95% CI 29.2,61.9), followed by pharmacies (25.4%;95% CI 13.5,42.3), private healthcare services (14.5%; 95% CI 6.3,30.0), international organizations (12.0%; 95% CI 5.6,24.1), and civil society organizations (3.1%; 95% CI 0.9,9.7) (results not shown). G-G that used health services in Mexico had a mean age of 34.2 years (95% CI 30.6,37.9). Most were men (72.8%; 95% CI 55.9,84.9) who worked during their last stay in Mexico (59.8%; 95% CI 45.6,72.5); 78.9% (95% CI 59.7,90.4) in the agriculture/livestock, industry, or construction sectors. The majority reported crossing to Mexico at least once a month (62.1%; 95% CI 40.9,79.6). The mean number of days spent in Mexico was 41.9 days (95% CI 27.0,56.8) overall.

Table 4 describes the factors associated with the cross-border use of health services by G-G only, given the small sample size of the other groups. The crude odds ratio of the association between having worked on their last visit to Mexico and cross-border use of health services was 3.02 (95% CI 1.23,7.45), and the crude odds ratio of the association between having worked in agriculture/livestock, industry, or construction in their last stay in Mexico, as compared to other sectors, and cross-border use of health services was 16.39 (95% CI 2.36,112.38). The bivariate analyses showed no association between age, sex, ethnicity, education, days spent in Mexico in the last visit, most recent health problem being a wound or accident, and cross-border use of health services (Table 4).

**Table 4:** Factors associated with cross-border use of health services by Guatemalans living in Guatemala.

	Crude OR (95% CI)	Model 1 Adjusted OR (95% CI)	Model 2^1^ Adjusted OR (95% CI)
	n = 100	n = 100	n = 53
**Work status in their last stay in Mexico**			-
Did not have a job/not working	REF.		
Had a job/worked	3.02* (1.23,7.45)	3.45* (1.03, 11.65)	-
**Type of work performed during their last stay in Mexico**			
Work not related to agriculture/livestock, industry, or construction	REF.		
Working in agriculture/livestock, industry, or construction	16.39** (2.36,112.38)	-	26.67* (1.97,360.85)
**Age **	0.99 (0.99,1.04)	1.00 (0.95,1.06)	1.00 (0.94,1.08)
**Sex** Female	Ref.		
Male	0.46 (0.18,1.13)	1.04 (0.27,4.07)	0.37 (0.04,3.75)
**Ethnicity** Not indigenous	Ref.		
Indigenous	1.38 (0.58,3.26)	1.93 (0.63,5.92)	5.48 (0.21,144.98)
**Education**			
At least some formal education	REF.		
No formal education	2.5 (0.49,12.57)	2.58 (0.49,13.48)	1.42 (0.13,15.65)
**Days spent in Mexico on the last visit**	1.0 (0.99,1.02)	0.99 (0.98,1.01)	0.99 (0.98,1.01)
**Most recent health problem**			
Health problems different from wounds or accident	REF.		
Wound or accident	2.33 (0.82,6.60)	2.03 (0.64,6.45)	0.73 (0.08,6.32)

^1^ Restricted to those who had worked in Mexico

In the multivariable models in Table 4, Model 1 includes work status as an independent variable, and Model 2 includes work sector as an independent variable and is therefore restricted to participants who worked in Mexico. We found that among participants who worked in Mexico, the odds of cross-border use of health services was 3.45 (95% CI 1.02,11.65) times the odds among participants who did not work (Table 4, Model 1). We also found that among participants who worked in the agriculture/livestock, industrial, or construction sectors, the odds of cross-border use of health services was 26.67 (95% CI 1.97,360.85) times the odds among participants who worked in other sectors (e.g., merchants, informal commerce, domestic service) (Table 4, Model 2).

## Discussion

The goal of this analysis was to describe the cross-border use of healthcare services and associated factors among people crossing the border between Mexico and Guatemala. Of the diverse profiles of persons that move through this busy border, according to our results the group that more frequently engaged in cross-border use of health services were male adults, born and living in Guatemala, who crossed the border with Mexico frequently to work in the agricultural/livestock, industrial, or construction sectors.

Overall, survey participants reported a considerably lower prevalence of recent health problems (2.6%) in comparison to the one reported for the general adult population (18+ years) in Chiapas, Mexico (16.7%), the main state bordering Guatemala, as calculated by the authors with data from Mexico’s General Directorate of Epidemiology [21]. The difference could be due to sampling differences among adult populations living in Chiapas, specifically differences among age, sex, and educational and socioeconomic groups, as well as the “healthy migrant” effect [22]. We could not find data on the prevalence of any health need in the past weeks or months in in the Guatemalan population, but the more frequent morbidities for seeking care in the general Guatemalan population (acute respiratory infections, urinary tract, and digestive problems) [23] are similar to the ones in our sample, with the exception of wound and accidents being the most common need in our study. Further, we found that M-M had a significantly lower prevalence of reported recent health problems compared to G-G and G-M, even though M-M were older. This could be explained by the different living and working conditions of these groups, but further investigation is required.

The main health problems reported in our sample differed from those reported in the National Health and Nutrition Survey in Mexico (ENSANUT). In our sample, the main recent health problem was wounds and accidents, whereas in Chiapas, other non-specified health problems (42.0%) and chronic diseases (18.4%) were the main health issues [24]. Additionally, only 1.7% of the adult population reported having had an accident in the past two weeks. However, our findings are consistent with studies in the U.S. among immigrant farmworkers, where work-related hazards are also among the main health problems reported [25, 26]. Since our sample was composed mainly of agricultural/livestock migrant workers, the aggregated results mostly represent the health problems of these groups.

As for the location of healthcare services sought by our survey population, most participants received care in public facilities. Given the specific question asked in our survey, it is not possible to know if the services used were those provided by the Ministry of Health, which serves populations without publicly available medical insurance, or by another public institution providing services through social security. However, a study conducted in Mexico among Guatemalan workers identified that 97.1% of participants did not have a signed employment contract, and only 2.0% had medical insurance as a work benefit [27]. This leads us to believe that the main type of public health services used by participants in Mexico may have been those offered by the Ministry of Health. Future studies should delve deeper into the type of healthcare facilities used, services offered, and out-of-pocket costs associated with care.

We also found that the use of pharmacy-based clinics by G-G participants (17.8%), was similar to that of the adult population of Chiapas, Mexico (11.1%) as based on data from Mexico’s National Survey on Nutrition and Health 2018, but much lower than the one of G-M participants (48.1%). The higher use of pharmacy-based clinics among G-M participants could be due to the fact that pharmacies in Mexico have adjacent doctors’ offices as part of the services they offer, which has accelerated between 2012 and 2018 [28]. The main reasons reported for using pharmacy-based services are the availability of services and drugs and shorter wait times [28]. Another reason for the use of pharmacy-based services could be related to cost: a search in the websites of pharmacies in Mexico shows that most of them charge around $1.50 USD for a doctors’ visit, compared to $25.00-$50.00 USD in other private settings. Observed differences between G-M and G-G could also be associated with the presence of more serious health problems among the latter group, which need to be resolved in a hospital versus a pharmacy-based health clinic.

Regarding the cross-border use of health services, our survey had two main findings: 1) Health-related factors were not the main reason for crossing; and 2) G-G was the only population group in our study that reported cross-border use of health services. Since the main reason for crossing the border among G-G was work-related, this leads us to hypothesize that most cross-border use of health services by Guatemalans in Mexico is circumstantial, meaning that it is caused by a health need that happened during a visit to Mexico due to other reasons (i.e. employment). In this sense, the main driver of cross-border use in this population seems to be different than the ones more frequently reported in the literature (distance, familiarity, cost or availability of specialized care) [1, 2, 12].

While did not ask about the migration status of participants, the Emif-Sur survey indicates that about three quarters (74%) of Guatemalans working in Mexico do so with some type of visa, but only 3.1 of them have a contract [29]. This is important in terms of access to health services, meaning that Guatemalan workers in Mexico could have limited access to services because of migration- and employment-related issues.

As for the first finding, only 1.0% of the total participants stated that the reason for their last cross to a country where they were not living was health-related. To our knowledge, there are no previous studies conducted on the border between Mexico and Guatemala that analyze this phenomenon in a representative sample, and these findings suggest the need to further explore it. Previous studies on other highly dynamic borders have shown that crossing the border for health-related factors is fairly common, with a recent survey in the Mexico-US land border reporting that one-fifth of all crossings to Mexico of persons living in the US were caused by health-related activities (seeking medical or dental care, or buying medicines) [30]. Another related finding is that M-M represented the highest percentage of crossings due to health-related reasons. This is similar to what has been reported for the U.S.-Mexico border, where people living in the U.S. are more likely to cross into Mexico for health-related reasons than the other way around [6, 7]. Since no M-M who had a recent health problem reported receiving healthcare in Guatemala, a possible interpretation of this finding could be that the main health-related reason for their crossing was to buy medicines. Future studies should include more questions regarding health-related border-crossings, such as specific health-related reasons for the crossing.

As for the second finding, 65.4% of G-G who had a recent health need received care in Mexico. The cross-border use of health services among G-G was associated with being migrant workers, and related to the agricultural/livestock, industrial and construction sectors compared to others. Based on these findings, as well as on the main type of health needs reported (i.e., wounds and accidents), perhaps G-G does not cross the border with the aim of gaining access to healthcare services. Instead, they travel to Mexico for reasons and access health care there upon experiencing an acute health issue. This finding is relevant, since using healthcare services while working in a country outside the country of residence is not, at present, part of the definition of cross-border use of health services [1]. Further studies are needed to identify if this type of use is based on preference or urgency of care.

Our survey had four main limitations. First, since this was a cross-sectional survey, we can only state associations between the use of cross-border health services and the employment status and type of work performed. Second, due to the time-venue sampling design, there is a possibility that the same person was surveyed more than once. However, it is unlikely that this happened based on the short time in which the survey was collected. Third, it is not possible to analyze the specific health-related reason that motivated participants’ travel because the survey answer choice combined several reasons in the same option (going to the doctor, buying medicines, or other health-related reasons). Finally, the number of G-M, M-M, and M-G that had a health problem and accessed health services was too small to understand their health-related border-crossing behaviors and hindered us from being able to make comparisons with the G-G group regarding these outcomes. On the other hand, people crossing the Mexico-Guatemala border employ both official ports of entry and non-official crossings [31]. The points where the survey was conducted are located close, but not adjacent, to the ports of entry, so participants may include persons coming through either official or non-official crossings.

## Conclusions

To our knowledge, this is the first report on the cross-border use of health services on the Mexico-Guatemala border. Findings suggest that the cross-border use of healthcare services is infrequent. Yet, Guatemalans make use of services in Mexico when traveling for work-related or other activities. Cross-border use of health services among temporary agricultural, industrial, or construction workers is mainly associated with wounds and accidents, and could be due, in part, to work-related injuries. The latter points to the importance of considering the health needs of temporary, transborder workers in health policies and developing strategies to facilitate and increase their access to healthcare.
